# Glioblastomas with primitive neuronal component harbor a distinct methylation and copy-number profile with inactivation of TP53, PTEN, and RB1

**DOI:** 10.1007/s00401-021-02302-6

**Published:** 2021-04-19

**Authors:** Abigail K. Suwala, Damian Stichel, Daniel Schrimpf, Sybren L. N. Maas, Martin Sill, Hildegard Dohmen, Rouzbeh Banan, Annekathrin Reinhardt, Philipp Sievers, Felix Hinz, Mirjam Blattner-Johnson, Christian Hartmann, Leonille Schweizer, Henning B. Boldt, Bjarne Winther Kristensen, Jens Schittenhelm, Matthew D. Wood, Guillaume Chotard, Rolf Bjergvig, Anirban Das, Uri Tabori, Martin Hasselblatt, Andrey Korshunov, Zied Abdullaev, Martha Quezado, Kenneth Aldape, Patrick N. Harter, Matija Snuderl, Jürgen Hench, Stephan Frank, Till Acker, Sebastian Brandner, Frank Winkler, Pieter Wesseling, Stefan M. Pfister, David E. Reuss, Wolfgang Wick, Andreas von Deimling, David T. W. Jones, Felix Sahm

**Affiliations:** 1 Department of Neuropathology, Institute of Pathology, Heidelberg University Hospital, Heidelberg, Germany; 2 Clinical Cooperation Unit Neuropathology, German Cancer Research Center (DKFZ), German Consortium for Translational Cancer Research (DKTK), Heidelberg, Germany; 3 Department of Neurological Surgery, Helen Diller Research Center, University of California San Francisco, San Francisco, CA USA; 4 Department of Pathology, University Medical Center Utrecht, Utrecht University, Utrecht, The Netherlands; 5 Hopp Children’s Cancer Center (KiTZ), Heidelberg, Germany; 6 Division of Pediatric Neurooncology, German Cancer Research Center (DKFZ), German Cancer Consortium (DKTK), Heidelberg, Germany; 7 Institute of Neuropathology, University of Giessen, Giessen, Germany; 8 Pediatric Glioma Research Group, German Cancer Research Center (DKFZ), Heidelberg, Germany; 9 Department of Neuropathology, Institute of Pathology, Hannover Medical School, Hannover, Germany; 10 Department of Neuropathology, Berlin Institute of Health, Charité-Universitätsmedizin Berlin, Corporate Member of Freie Universität Berlin, Humboldt-Universität zu Berlin, Berlin, Germany; 11 German Cancer Consortium (DKTK), Partner Site Berlin, German Cancer Research Center (DKFZ), Heidelberg, Germany; 12 Department of Pathology, Odense University Hospital, Odense, Denmark; 13 Department of Clinical Research, University of Southern Denmark, Odense, Denmark; 14 Department of Pathology, Rigshospitalet, Copenhagen University Hospital, Copenhagen, Denmark; 15 Department of Clinical Medicine and Biotech Research and Innovation Center (BRIC), University of Copenhagen, Copenhagen, Denmark; 16 Institute of Pathology and Neuropathology, Department of Neuropathology, University Hospital Tübingen, Tübingen, Germany; 17 Department of Pathology, Oregon Health and Science University, Portland, OR USA; 18 Department of Pathology, Hospital Center University of Bordeaux, Bordeaux, France; 19 Department of Biomedicine, University of Bergen, Bergen, Norway; 20 Division of Haematology/Oncology, The Hospital for Sick Children, 555 University Ave, Toronto, ON M5G 1X8 Canada; 21 The Arthur and Sonia Labatt Brain Tumour Research Centre, The Hospital for Sick Children, Toronto, Canada; 22 Department of Medical Biophysics, Faculty of Medicine, University of Toronto, Toronto, Canada; 23 Institute of Neuropathology, University Hospital Münster, Munster, Germany; 24 Laboratory of Pathology, National Cancer Institute Centre for Cancer Research, Bethesda, MD USA; 25 Neurological Institute (Edinger Institute), Goethe-University Frankfurt am Main, Frankfurt am Main, Germany; 26 Frankfurt Cancer Institute (FCI), Frankfurt am Main, Germany; 27 German Cancer Research Center (DKFZ), Heidelberg, Germany; 28 Division of Neuropathology, NYU Langone Health, New York, USA; 29 Laura and Isaac Perlmutter Cancer Center, NYU Langone Health, New York, USA; 30 Division of Molecular Pathology and Diagnostics, NYU Langone Health, New York, USA; 31 Division of Neuropathology, Institute of Pathology, Basel University Hospital, Basel, Switzerland; 32 Division of Neuropathology, The National Hospital for Neurology and Neurosurgery, University College London Hospitals, London, UK; 33 Clinical Cooperation Unit Neurooncology, German Consortium for Translational Cancer Research (DKTK), German Cancer Research Center (DKFZ), Heidelberg, Germany; 34 Department of Neurology and Neurooncology Program, National Center for Tumor Diseases, Heidelberg University Hospital, Heidelberg, Germany; 35 Princess Máxima Center for Pediatric Oncology, Utrecht, The Netherlands; 36 Department of Pathology, Amsterdam University Medical Centers/VUmc and Brain Tumor Center Amsterdam, Amsterdam, The Netherlands; 37 Department of Pediatric Oncology, Hematology and Immunology, University Hospital Heidelberg, Heidelberg, Germany; 38 Department of Neurodegenerative Disease, UCL Queen Square Institute of Neurology, Queen Square, London, UK

**Keywords:** GBM, PNET, DNA methylation, Phenotype, Classification, Plasticity

## Abstract

Glioblastoma IDH-wildtype presents with a wide histological spectrum. Some features are so distinctive that they are considered as separate histological variants or patterns for the purpose of classification. However, these usually lack defined (epi-)genetic alterations or profiles correlating with this histology. Here, we describe a molecular subtype with overlap to the unique histological pattern of glioblastoma with primitive neuronal component. Our cohort consists of 63 IDH-wildtype glioblastomas that harbor a characteristic DNA methylation profile. Median age at diagnosis was 59.5 years. Copy-number variations and genetic sequencing revealed frequent alterations in *TP53*, *RB1* and *PTEN,* with fewer gains of chromosome 7 and homozygous *CDKN2A/B* deletions than usually described for IDH-wildtype glioblastoma. Gains of chromosome 1 were detected in more than half of the cases. A poorly differentiated phenotype with frequent absence of GFAP expression, high proliferation index and strong staining for p53 and TTF1 often caused misleading histological classification as carcinoma metastasis or primitive neuroectodermal tumor. Clinically, many patients presented with leptomeningeal dissemination and spinal metastasis. Outcome was poor with a median overall survival of only 12 months. Overall, we describe a new molecular subtype of IDH-wildtype glioblastoma with a distinct histological appearance and genetic signature.

**Supplementary Information:**

The online version contains supplementary material available at 10.1007/s00401-021-02302-6.

## Introduction

Glioblastoma is the most common malignant brain tumor among adults. At present, median overall survival after standard of care treatment, which includes resection followed by radio- and chemotherapy, is only 15 months [15]. This tumor type is predominantly found in the elderly, with a peak of incidence at 75–84 years [23]. Compared with other solid tumors, metastases are rare, and patients mainly die from neurological complications [31]. However, leptomeningeal dissemination and spinal metastasis, which are more common in patients with embryonal tumors such as medulloblastoma, may occur in a minor fraction of glioblastoma patients [19].

Morphologically, glioblastoma may present with different histological features. Presence of microvascular proliferation and/or necrosis, often accompanied by perinecrotic palisades, are mandatory for the histological diagnosis of glioblastoma. Irrespective of these characteristics, glioblastoma may harbor histological patterns of small cells, primitive neuronal cells, oligodendroglial components, gemistocytes, granular cells, lipidized cells, multinucleated giant cells and sarcomatous components. Such histological patterns occurring in small areas within an otherwise ‘typical’ case are separated from true histological variants, which are defined by the clear predominance of a given pattern in one tumor. Giant-cell glioblastoma, gliosarcoma and epithelioid glioblastoma are listed as variants in the WHO classification of tumors of the central nervous system (CNS) from 2016. Except for giant-cell glioblastoma, which is reported to have a tendency towards a better prognosis, different histological features are not associated with difference in survival [18].

In 2009, Perry et al. described 53 brain tumors with combined features of malignant gliomas and primitive neuroectodermal tumors. Common findings were loss of GFAP expression, p53 and synaptophysin expression, highly elevated Ki-67 index, MYC/MYCN amplification in the primitive neuronal component, a poor clinical prognosis and increased risk of leptomeningeal dissemination. A fluorescence in-situ hybridization (FISH) analysis revealed evidence that this group of tumors generally represents conventional gliomas from which embryonal-like elements with neuronal immunophenotypes secondarily emerged [25]. The group was included in 2016 WHO classification of tumors of the CNS and is now referred to glioblastomas with primitive neuronal component.

Besides histological patterns, glioblastomas can be divided into different methylation subgroups. Isocitrate dehydrogenase (IDH)-wildtype glioblastoma diagnosed in adults mainly fall into one of the following subgroups: mesenchymal, or receptor tyrosine kinase (RTK) 1 or 2 [5, 33]. DNA methylation-based classification was shown to correlate with previous molecular classes of glioblastoma described by the The Cancer Genome Atlas (TCGA) [22, 36] and is a helpful tool to define a precise diagnosis, especially if histology is not compelling or material is limited [5, 6, 8]. For some entities such as medulloblastoma and meningioma, methylation profiling is also of prognostic relevance [21, 29]. In addition, copy-number profiles calculated from DNA-methylation profiling can help to define a precise diagnosis [27]. Based on cIMPACT-NOW update 3, diffuse IDH-wildtype gliomas that are histologically lower grade (WHO grade 2 or 3) can be diagnosed as IDH-wildtype glioblastoma based upon presence of epidermal growth factor receptor (*EGFR)*-amplification and/or promoter mutation in telomerase reverse-transcriptase (*TERT*) and/or a combination of gain of complete chromosome 7 and loss of complete chromosome 10 [4].

Here, we report a novel methylation subgroup of IDH-wildtype glioblastoma that differs from known molecular subgroups in terms of methylation and copy-number profile and is linked to the histological variant of glioblastomas with primitive neuronal component.

## Material and methods

### Tissue samples

Samples were collected from neuropathology departments from multiple medical centers. Case selection was performed through t-distributed stochastic neighbor embedding (*t*-SNE) analysis of genome-wide DNA methylation data in a cohort of more than 75,000 tumors. Group formation was based on similarities in DNA methylation profiles. Tissue and data collection were performed in accordance with local ethics regulations and approval.

### DNA methylation and t-SNE analysis

DNA extraction was performed as previously described [35]. DNA methylation profiles were generated using the Infinium HumanMethylation450 (450 k) or Infinium MethylationEPIC (850 k) BeadChip array (Illumina, San Diego, USA) according to the manufacturer’s instructions. The data were processed as previously described [5]. The *t*-SNE plot was calculated using the 15,000 most variable CpG sites according to standard deviation, 3,000 iterations and a perplexity value of 10. Reference cases were selected based on a high classifier score (> 0.9) in the brain tumor classifier. Methylation of the O^6^-alkylguanine DNA alkyltransferase (*MGMT*) promoter region was calculated as described previously [35].

### Copy-number plots

To generate copy-number variation (CNV) profiles from the methylation array data we used the ‘conumee’ package in R (http://bioconductor.org/packages/release/bioc/html/conumee.html, https://github.com/dstichel/conumee) with additional baseline correction. By condensing multiple copy-number plots, summary CNV plots were created.

### Gene sequencing and mutational burden

Next-generation sequencing was performed on a NextSeq 500 sequencer (Illumina) as described previously [28]. Exonic and splicing indels and nonsynonymous single-nucleotide variants (SNVs) with a frequency of ≤ 0.001 in the 1000 genomes database (https://www.internationalgenome.org/) were identified after subtracting low-quality calls. Telomerase reverse-transcriptase (*TERT)*-promoter mutations were extracted and evaluated separately due to the often low number of reads at this position.

### Immunohistochemistry

Immunohistochemistry was performed on 3 µm thick FFPE tissue sections attached on StarFrost Advanced Adhesive slides (Engelbrecht, Kassel, Germany), followed by drying at 80 °C for 15 min and conducted on a BenchMark Ultra immunostainer (Ventana Medical Systems, Tucson, USA). Slides were pretreated with Cell Conditioning Solution CC1 (Ventana Medical Systems) for 32 min at room temperature. Primary antibodies were incubated at 37 °C for 32 min. Afterwards we used Ventana standard signal amplification, UltraWash, counter-staining with one drop of hematoxylin for 4 min, and one drop of bluing reagent for 4 min. For visualization ultraView Universal DAB Detection Kit (Ventana Medical Systems) was used. Primary antibodies were diluted as followed: Glial fibrillary acidic protein (GFAP, 1:2000, Cell signalling, Promega), cytokeratin AE1/3 (RTU, DCS Hamburg, Germany), thyroid transcription factor 1 **(**TTF1, 1:50, clone EP229, Cell Marque, Rocklin, USA and 1:50, clone 8G7G3/1, Dako, Santa Clara, USA), tumor protein 53 (p53, 1:50, Novocastra, Wetzlar, Germany), synaptophysin (1:50, Cell Marque), retinoblastoma protein (Rb, 1:50, clone Ab-780, Merck, St. Louis, USA), NeuN (1:100, Milipore, Burlington, USA), neuron-specific enolase (NSE, 1:4, Linaris, Dossenheim, Germany), Ki67 (1:100, Dako). Stained slides were scanned on the Aperio AT2 Scanner (Aperio Technologies, Vista, USA) and digitalized using Aperio ImageScope software v12.3.2.8013.

### Statistical analysis

**Fig. 1:**
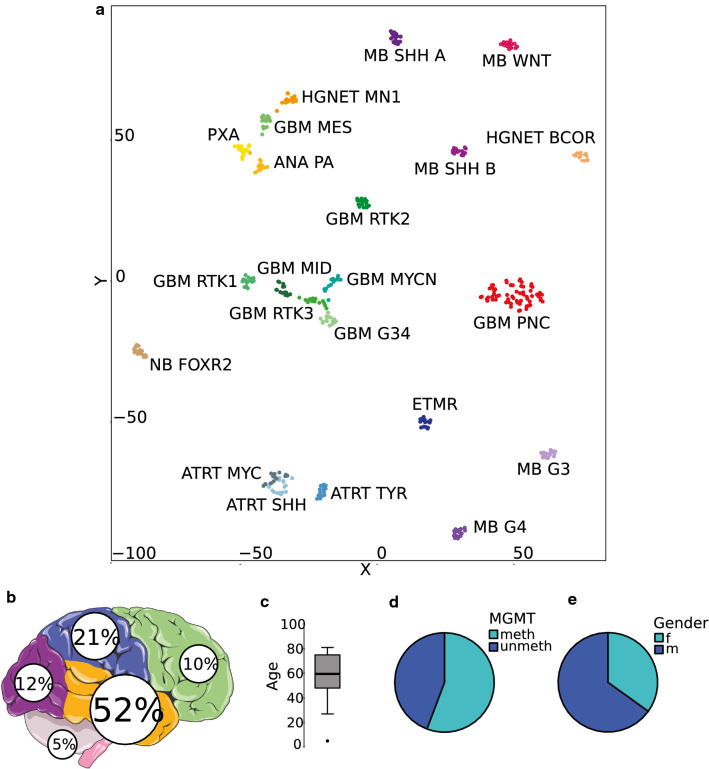
Glioblastomas with primitive neuronal component have a distinct methylation profile. **a** *t*-SNE analysis of 63 glioblastomas with primitive neuronal component and 311 reference cases. Distribution of primary localization **b**, age **c**, *MGMT* promoter methylation status **d** and gender **e** in glioblastomas with primitive neuronal component. *GBM PNC* glioblastoma with primitive neuronal component, *MB* medulloblastoma, *NB* neuroblastoma, *HGNET* high-grade neuroepithelial tumor, *ANA PA* anaplastic astrocytoma with piloid features, *PXA* pleomorphic xanthoastrocytoma, *(un)meth* (un)methylated

Sample sizes (*n*) are indicated in figures and figure legends. Kaplan–Meier curves were created and log-rank tests calculated using SPSS (IBM, Armonk, USA). For Fig. 1b, an image from Servier Medical Art (http://smart.servier.com/) was used with little modifications licensed under a Creative Commons Attribution 3.0 Unported License.

## Results

### Glioblastomas with primitive neuronal component have a distinct methylation profile

In a *t*-SNE analysis with more than 75,000 tumor samples, mainly including brain tumors and sarcomas, a distinct methylation class consisting of 63 samples was found. The group was located separate, yet close to the large but heterogeneous group of IDH-wildtype glioblastoma (data not shown). A more focused *t*-SNE analysis with selected cases of several reference comparison groups was conducted, highlighting the distinctiveness of the new molecular group (Fig. 1a). By looking at clinical data, we discovered that more than half of the tumors were localized in the temporal lobe (Fig. 1b). Patients were mostly older adults, with a median age of 59.5 years [5–81 years] (Fig. 1c). For 55.7% (34/61) of samples the promoter region of the *MGMT* gene was methylated (Fig. 1d). There was a predominance of males [1.7:1] (Fig. 1e). Almost all samples were obtained at first surgery for the brain tumor; whereas, one sample was diagnosed as a recurrence of an IDH-wildtype glioblastoma. Interestingly, the primary tumor of this case as well as two recurrent tumors from another case clustered within known methylation groups of IDH-wildtype glioblastoma (Suppl. Figure 1).

### Frequent alterations in TP53, PTEN and RB1

**Fig. 2:**
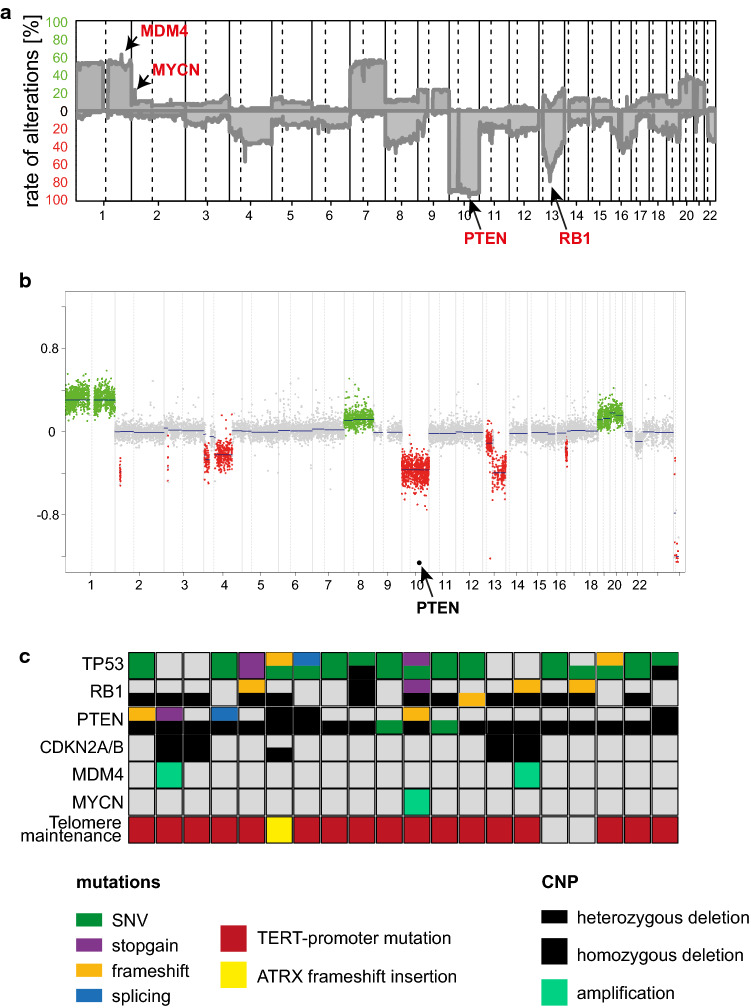
Frequent alterations in *TP53*, *PTEN* and *RB1*. **a** Summation copy-number plot of 63 glioblastomas with primitive neuronal component. **b** Copy-number plot of a glioblastoma with primitive neuronal component showing homozygous *PTEN* deletion. **c** Mutations and copy-number alterations in selected genes detected in 20 samples

Closer inspection of the copy-number profiles revealed loss of chromosome 10 in more than 90 percent of cases; whereas, a gain of chromosome 7 was only detected in half of the samples (Fig. 2a). Twelve cases (19%) presented with homozygous phosphatase and tensin homolog (*PTEN)* deletion (Fig. 2b). Loss of chromosome 13 was detected in 54.0% (34/63) of samples. Focal homozygous retinoblastoma tumor suppressor gene 1 (*RB1)* deletion was present in seven cases (11.1%). Interestingly, half of the cases showed a gain of chromosome 1. *MDM2* and *MDM4* were each focally amplified in 6.3% of cases (4/63). *MYCN* amplification was present in 14.3% (9/63) with two cases amplified for *MYC* (3.2%). Homozygous cyclin-dependent kinase inhibitor (*CDKN) 2A/B* deletion was only detected in 15.9% (10/63).

Panel sequencing was performed for 19 cases. Another case was retrieved from TCGA with exome-sequencing data available. *TERT*-promoter mutations were found in 17 of 20 samples. In one case without *TERT*-promoter mutation an alpha thalassemia/mental retardation syndrome, X-linked (*ATRX)*-frameshift insertion was detected. In 80% of samples (16/20) at least one *TP53* mutation was found. Interestingly, of nine cases where only one *TP53* mutation was detected, seven showed a clear enrichment of the mutated allele; whereas, only two of these samples presented with a heterozygous loss of the *TP53* locus (suggesting copy-neutral LOH as a prominent mechanism for loss of the wildtype allele). The four cases without detected *TP53* mutations or deletions all showed homozygous deletion of the *CDKN2A/B* locus and two cases additionally had a clear amplification of *MDM4.* Seven cases showed a *PTEN* mutation, accompanied by heterozygous chromosomal deletion of the *PTEN* locus in five of these cases. *RB1* mutations were detected in five samples, with four of these cases displaying a heterozygous deletion of the genetic locus (Fig. 2c).

### Embryonal morphology, lack of GFAP expression and TTF1 positivity are common features

**Fig. 3:**
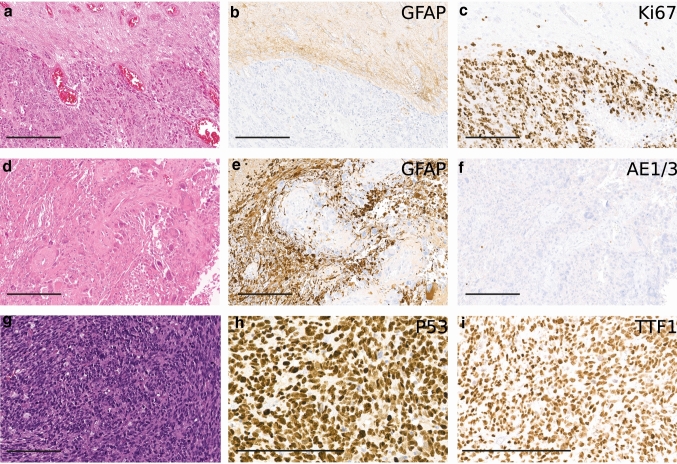
Histology of glioblastoma with primitive neuronal component. Cases present with high nuclear-to-cytoplasmic ratio and frequent mitoses in the areas with embryonal phenotype (**a**, **d**, **g**). GFAP expression is lacking in these tumor regions (**b**, **e**). Glial areas with retained GFAP expression **b**, **e** and lower proliferation index **c** can be sharply demarcated from GFAP negative areas. Lacking cytokeratin expression in GFAP negative areas (**f**), but nuclear positivity for p53 (**h**) and TTF1 (clone EP229) (**i**). Scale bar 200 µm

From 45 cases with known histological diagnosis, 28 cases were initially diagnosed as glioblastoma, five cases as gliosarcoma, 11 cases as primitive neuroectodermal tumor (PNET) and one case as ganglioneuroblastoma. Additional primitive neuronal component was described for seven of the histologically defined glioblastomas and five of the gliosarcomas. For three cases, neuroendocrine metastasis was considered as the diagnosis. Tumors showed undifferentiated, embryonal cells with high nuclear–cytoplasmatic ratio and high mitotic activity. However, none of the cases showed Homer Wright rosettes or clear cell wrapping that are characteristic features of medulloblastoma and other embryonal neoplasms. Some cases showed a biphasic pattern with additional glial tumor parts separated from embryonal tumor parts. For those samples that showed additional glial parts, these were mostly sharply separated from primitive parts; whereas, a minority of cases showed intermixed sections. Negative GFAP staining in embryonal appearing tumor cells was shown for 84% (21/25) of cases (Fig. 3). We additionally performed DNA-methylation profiling from only GFAP positive glial appearing tumor components of eight cases. Interestingly, three of the cases did not cluster with their GFAP-negative counterparts but to other methylation groups of IDH-wildtype glioblastoma (Suppl. Figure 1). Copy-number profiles of GFAP positive and negative tumor parts were mostly congruent. One case presented with a gain of chromosomal arm 1p only in the GFAP negative part. Another case harbored an amplification of the *MYCN* locus in GFAP negative areas; whereas, GFAP positive areas were *MYCN* wildtype. Almost all cases (27/29) presented with strong nuclear staining for p53 in the embryonal tumor component. Synaptophysin was positive for 85.7% (12/14) in embryonal tumor parts (Suppl. Figure 3). Tumors that stained negative for Synaptophysin showed positive staining for at least one other neuronal marker (NeuN or neuron specific enolase, NSE). All tumors tested showed positivity for NSE (9/9); whereas, only four out of eleven tumors were positive for NeuN (36.4%). We also stained for RB1 expression. However, none of the cases showed a clear loss of nuclear expression (Suppl. Figure 3). Surprisingly, 77.8% (14/18) cases showed positive staining of the nuclei of tumor cells for TTF1 using the EP229 clone (Fig. 3) in embryonal, GFAP-negative tumor components only. We additionally stained 20 conventional glioblastomas, 10 of which were small cell variants, that were all negative for the TTF1 EP229 clone. However, using the TTF1 8G7G3/1 clone only one of the samples showed nuclear positivity (1/15, 6.6%, Suppl. Figure 3). Potentially, further differences may also apply for other clones, and for findings on RB1 positivity, respectively.

### Leptomeningeal dissemination is frequent and survival is poor

**Fig. 4:**
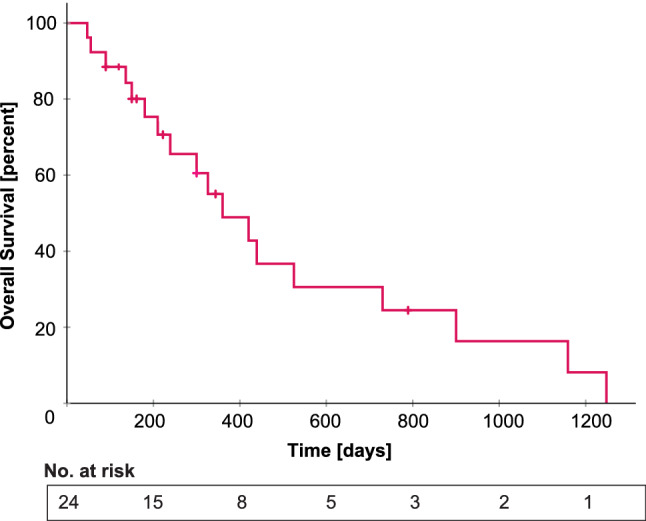
Overall survival in glioblastoma with primitive neuronal component. Median survival is 360 days

Survival data were available for 24 patients. Median overall survival was only 12 months (360 days, Fig. 4). There was no difference in survival regarding *MGMT* promoter methylation status (Suppl. Figure 2a). Progression-free survival was available for 23 patients and median PFS was 8 months (240 days, Suppl. Figure 2b). Interestingly, for 10 patients with clinical data available, leptomeningeal dissemination with spinal metastasis was reported in four patients. For one patient, the spinal metastasis was passed through DNA methylation analysis and showed a very similar methylation and copy-number profile as the primary tumor (Suppl. Figure 1). Three additional patients showed intracerebral metastases. Most patients were treated according to the Stupp protocol with fractionated irradiation and temozolomide.

## Discussion

In this work, we present a new molecular type that is linked to a specific histological pattern of IDH-wildtype glioblastoma. The tumors present with a highly undifferentiated morphology, frequent leptomeningeal dissemination and occasional sarcomatous features. Lack of GFAP expression and nuclear positivity for TTF1 are common in embryonal components which may cause misinterpretation as carcinoma although these tumors do not express cytokeratins. Clinical outcome is very poor and in line with other IDH-wildtype glioblastomas. For their focal resemblance we provisionally named these tumors glioblastomas with primitive neuronal component.

Glioblastomas with primitive neuronal component harbor a unique methylation profile that is clearly distinguishable from methylation (sub-)groups known so far in the v11b4 version of the brain tumor classifier [5]. In a *t*-SNE analysis with more than 75,000 reference cases the group clusters close to IDH-wildtype glioblastomas. One case was a recurrent tumor of an IDH-wildtype glioblastoma, subtype mesenchymal. Another case recurred as IDH-wildtype glioblastoma, subtype RTK2. For three samples, GFAP-positive areas within the same tumor also clustered to distinct subtypes of IDH-wildtype glioblastoma. These results show that the new molecular entity described in this work clearly belongs to the type of IDH-wildtype glioblastoma as also discovered by the FISH analyses performed by Perry et al. [25], but it also indicates that molecular subtypes among IDH-wildtype glioblastomas may vary upon progression and several subtypes might even be present in one manifestation of a tumor. This indicates a high degree of plasticity in glioblastoma cells which is not a common feature of other tumor types (where methylation profiles stay remarkably constant over time), as also recently suggested based on single-cell trancriptomic analyses [34]. Neftel et al. showed that IDH-wildtype glioblastoma consists of four different cellular states that are all capable of tumor initiation and transition into another state [20]. Our study demonstrates that plasticity in glioblastoma is not only detected in the transcriptome but can also be found on DNA methylation level, which was thought to be more robust [5].

According to the 2016 WHO classification of brain tumors of the central nervous system, IDH-wildtype glioblastomas show homozygous deletion of *CDKN2A/B* in approximately 60% [18]. *CDKN2A* encodes for two proteins, one is p16 that activates retinoblastoma protein (Rb) through inhibition of cyclin-dependent kinases (CDK) 4 and 6, the second protein is p14arf activating p53 by inhibiting MDM2 [17, 26]. In glioblastoma with primitive neuronal component, *CDKN2A/B* is deleted in only 15.9% of samples. The pathway seems to be affected further downstream by frequent *RB1* deletions and mutations as well as *TP53* mutations. Interestingly, *TP53* mutations are usually linked to IDH-mutations in astrocytic tumors and only found in 25–30% of IDH-wildtype glioblastoma [7, 18, 24, 37]. About 80% of glioblastomas with primitive neuronal component, however, have *TP53* mutations. Regarding samples with genetic sequencing data available, only the small fraction of tumors lacking *TP53* mutations harbored homozygous *CDKN2A/B* deletion. *TP53* mutations together with *PTEN* mutations were also found in a study recently performed by Xu et al. performing exon sequencing on 11 glioblastomas with primitive neuronal component. In addition, the authors found frequent mutations in *PIK3CA* and *PIK3R1*; whereas, an *RB1* mutation was only found in one case [38].

Mutational inactivation of p53 correlates with higher proliferation, invasion and a more stem-like phenotype [2, 13]. In our cohort, the proliferation index of the tumors was generally very high and a substantial proportion of patients presented with intracranial or even spinal leptomeningeal dissemination, which is otherwise described to be a rare event in glioblastoma [19]. Therefore, regular radiological screening of the neuroaxis should be considered subsequent to detection of this tumor type.

In our cohort, *TERT* promoter mutations and a chromosome 7/10 signature in at least half of the samples are shared findings with conventional IDH-wildtype glioblastoma and further imply the belonging to this entity. However, aberrations in *RB1* and *MYC/MYCN* as well as leptomeningeal dissemination are characteristics of embryonal tumors as medulloblastomas or atypical teratoid rhabdoid tumors. Histologically, half of the cases were diagnosed as either PNET or glioblastoma/gliosarcoma with primitive neuronal component. However, the diagnosis PNET itself became obsolete with the WHO classification of CNS tumors released in 2016 [18]. The latest WHO classification for CNS tumors which will be released in spring 2021 still recognizes glioblastomas with primitive neuronal component, and loss of GFAP expression, MYC/MYCN gene amplification, elevated Ki67 proliferation index and increased cerebrospinal fluid dissemination. The histological defined pattern has wide overlap with our molecular defined subtype and, therefore, we named our methylation group glioblastomas with primitive neuronal component [18, 25]. Gliosarcomas with primitive neuronal component have also been described before [12, 14, 30], and interestingly, glioblastomas with PNET phenotype have also been described in mouse models with inactivation of *p53*, *Rb*, and *Pten* in neural progenitors [10, 11]. The morphology can also be shared by IDH-mutant gliomas [32]. However, none of the tumors described in our study harbored a pathogenic IDH-mutation.

In addition, we detected positivity of tumor cell nuclei for TTF1 using the EP229 clone; whereas, conventional glioblastomas, including small cell variants, all stained negative for this clone. It is also worth noting that nuclear TTF1 expression was only detectable in embryonal patterns in our tumors; whereas, glial tumor components did not express the protein. TTF1 expression in glioblastoma has not been described for this TTF1 clone so far. In neuropathological routine, TTF1 immunohistochemistry is most often used to distinguish metastases of lung and thyroid cancers from primary brain tumors. Although nuclear TTF1 expression in glioblastoma is rare, reactivity may differ among different antibody clones [9]. We additionally stained glioblastomas with primitive neuronal component with the TTF1 8G7G3/1 clone shown to be negative in glioblastomas and only detected positive nuclear staining for TTF1 in one case. These findings indicate that not all TTF1 clones are equally applicable to diagnose glioblastomas with primitive neuronal component. TTF1 is known to be expressed during not only lung and thyroid morphogenesis but also during brain development [3, 16] which might lead to the assumption that positivity for this protein in glioblastomas might represent further evidence for a more primitive and undifferentiated phenotype. It might also be hypothesized that glioblastomas with primitive neuronal component have undergone “pan-cancer convergence” that was described by Balanis et al. for epithelial cancers that acquire *TP53* and *RB1* mutations [1]. However, TTF1 expression can also be found among low grade tumors as pituicytomas or spindle cell oncocytomas of the pituitary gland.

In conclusion we describe a molecular variant of glioblastoma with overlap to a histological pattern presented previously. Glioblastomas with primitive neuronal component are characterized by a distinct methylation profile in the component of embryonal phenotype. Our study extends the basis for molecular diagnostics of challenging diagnostic cases.

## Supplementary Information

Below is the link to the electronic supplementary material.

Supplementary file1 (PDF 179 kb)
