# Supplementary figures and images for: Glioblastomas with primitive neuronal component harbor a distinct methylation and copy-number profile with inactivation of TP53, PTEN, and RB1
Source: Acta Neuropathol. 2021 Apr 19;142(1):179–89. doi: 10.1007/s00401-021-02302-6 (PMC8217054; doi:10.1007/s00401-021-02302-6)

Supplementary Figure 1

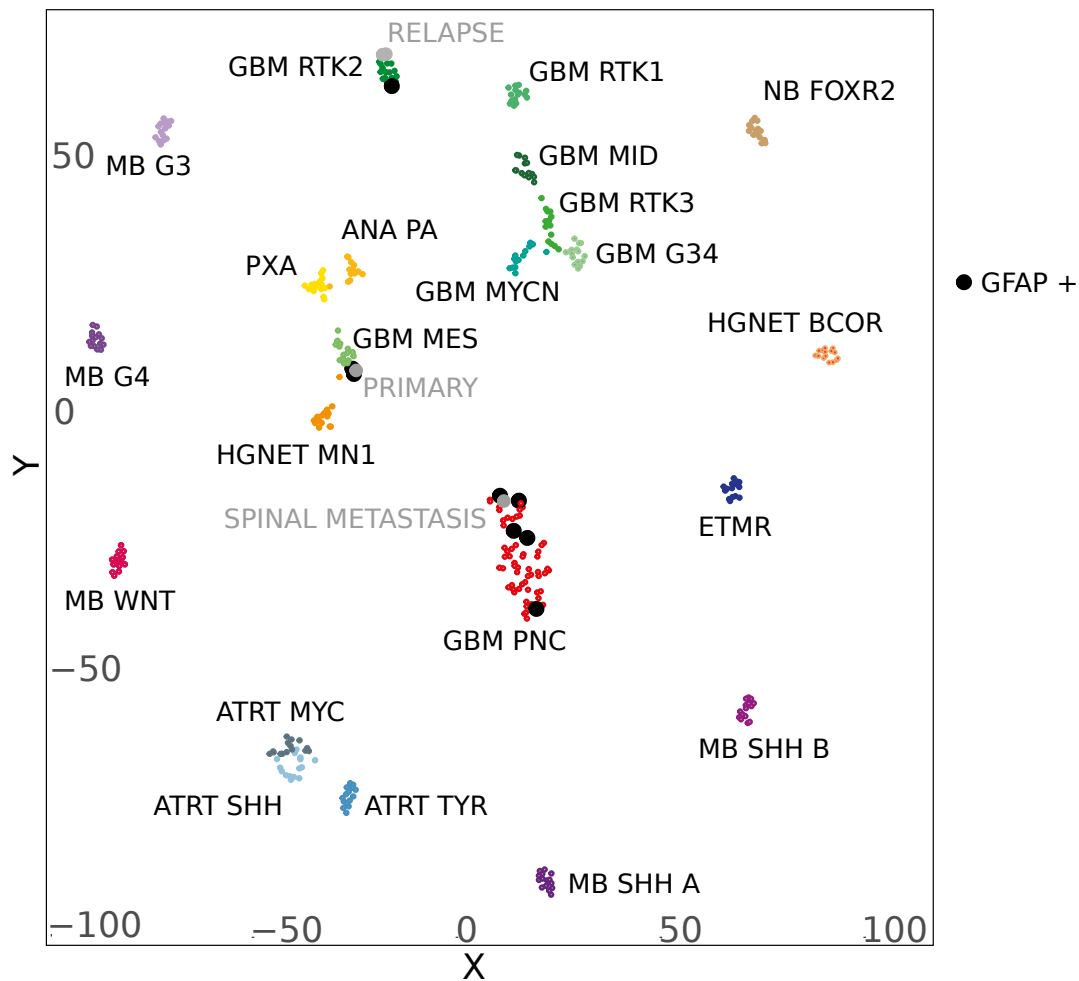

Supplementary Figure 2

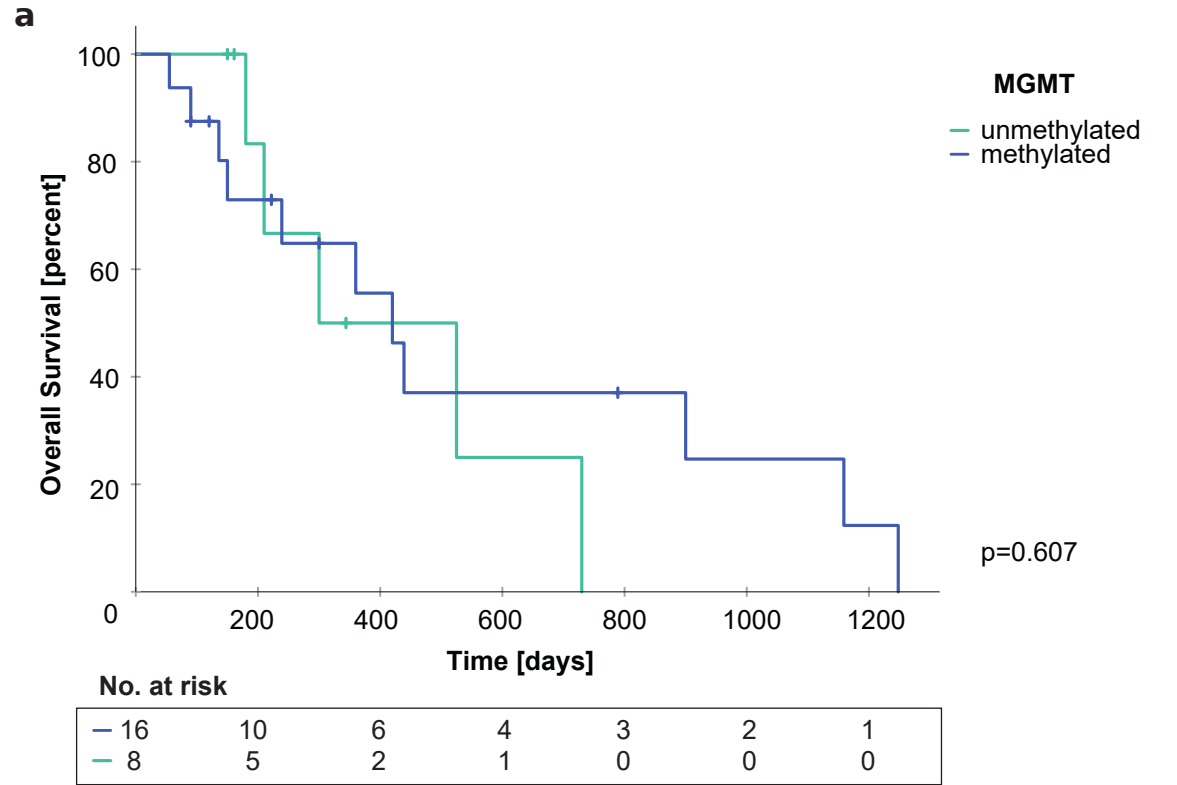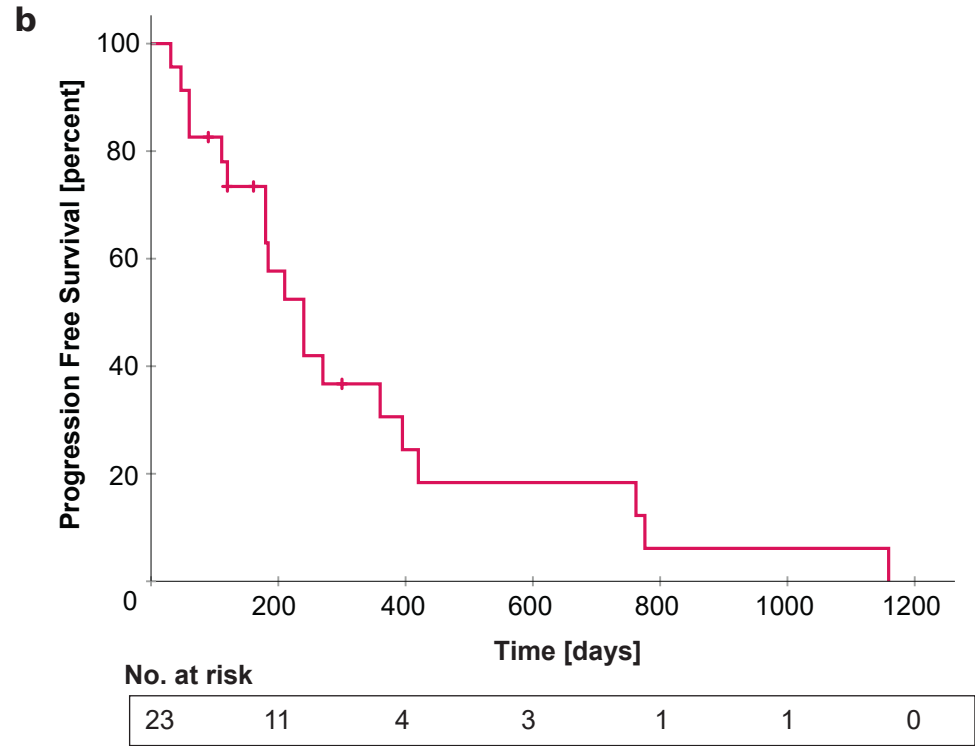

### Supplementary Figure 3

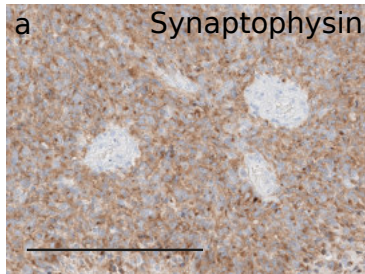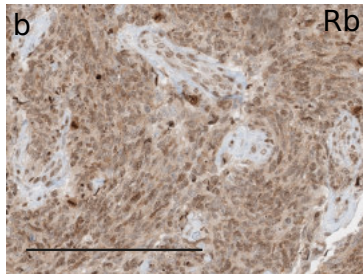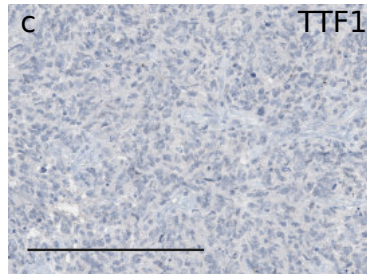

Supplement: Supplementary file 1 — Supplementary file1 (PDF 179 kb) [file 401_2021_2302_MOESM1_ESM.pdf]
